# Retraction: Astragaloside IV (AS-IV) alleviates the malignant biological behavior of hepatocellular carcinoma *via* Wnt/β-catenin signaling pathway

**DOI:** 10.1039/d4ra90047b

**Published:** 2024-05-02

**Authors:** ZhongYu Jiang, Zhen Mao

**Affiliations:** a Department of Cancer Center, Zhejiang Quhua Hospital Quzhou City Zhejiang Province 324004 China; b Department of Traditional Chinese Medicine, Gansu Provincial Hospital No. 204, Donggang West Road, Chengguan District Lanzhou City Gansu Province 730000 China maozhengssyy@163.com

## Abstract

Retraction of ‘Astragaloside IV (AS-IV) alleviates the malignant biological behavior of hepatocellular carcinoma *via* Wnt/β-catenin signaling pathway’ by ZhongYu Jiang *et al.*, *RSC Adv.*, 2019, **9**, 35473–35482, https://doi.org/10.1039/C9RA05933D.

The Royal Society of Chemistry hereby wholly retracts this *RSC Advances* article due to concerns with the reliability of the data in the published article.

The panels labelled ‘AIV 50 noml/l/Hep3B’ and ‘AIV 100 noml/l/Hep3B’ in Fig. 3b are duplicated in another publication by different authors (R. Sun, R. Zhai, C. Ma and W. Miao, *Cancer Med.*, 2020, **9**, 1141–1151).

Many panels in Fig. 4c have been duplicated in other publications by different authors (W. Xiao, Y. Liu, M. Dia, Y. Li, R. Peng, S. Yu and H. Liu, *Inter. Jour. Mol. Med.*, 2020, **46**, 700–708; T. Liu, A. Li, Y. Xu and Y. Xin, *Cancer Med.*, 2019, **8**, 751–760; and X. Wang, Y. Chang, M. Gao and F. Zhang, *Onco. Targets Ther.*, 2020, **13**, 10097–10109).

The ‘AIV (50 mg kg^−1^)/VEGF panel in Fig. 6c is duplicated in another publication by different authors (N. Li, C. Cheng and T. Wang, *Onco. Targets Ther.*, 2020, **21**, 4495–4505).

The authors were asked to provide the raw data for this article, but did not respond. Given the significance of the concerns about the validity of the data, and the lack of raw data, the findings in this paper are not reliable.

The authors have been informed but have not responded to any correspondence regarding the retraction.

Signed: Laura Fisher, Executive Editor, *RSC Advances*

Date: 17/04/2024

## Supplementary Material

